# Moderate nitrogen application facilitates Bt cotton growth and suppresses population expansion of aphids (*Aphis gossypii*) by altering plant physiological characteristics

**DOI:** 10.3389/fpls.2024.1328759

**Published:** 2024-03-06

**Authors:** Lixiang Guo, Lin Niu, Xiangzhen Zhu, Li Wang, Kaixin Zhang, Dongyang Li, Punniyakotti Elumalai, Xueke Gao, Jichao Ji, Jinjie Cui, Junyu Luo

**Affiliations:** ^1^ Zhengzhou Research Base, National Key Laboratory of Cotton Bio-breeding and Integrated Utilization, School of Agricultural Sciences, Zhengzhou University, Zhengzhou, China; ^2^ National Key Laboratory of Cotton Bio-breeding and Integrated Utilization, Institute of Cotton Research, Chinese Academy of Agricultural Sciences, Anyang, China; ^3^ Western Agricultural Research Center, Chinese Academy of Agricultural Sciences, Changji, China

**Keywords:** nitrogen fertilizer, aphids, Bt cotton, Bt toxin protein, soluble sugar

## Abstract

**Introduction:**

Excessive application of nitrogen fertilizer in cotton field causes soil and water pollution as well as significant increase of aphid population. Reasonable fertilization is an important approach to improve agricultural production efficiency and reduce agriculture-derived pollutions. This study was aimed to explore the effects of nitrogen fertilizer on the Bt cotton physiological characteristics and the growth and development of *A. gossypii*, a sap-sucking cotton pest.

**Methods:**

Five different levels of Ca(NO_3_)_2_ (0.0 g/kg, 0.3 g/kg, 0.9 g/kg, 2.7 g/kg and 8.1 g/kg) were applied into vermiculite as nitrogen fertilizer in order to explore the effects of nitrogen fertilizer on the growth and development of Bt cotton and aphids.

**Results:**

The results showed that the medium level of nitrogen fertilizer (0.9 g/kg) effectively facilitated the growth of Bt cotton plant and suppressed the population expansion of aphids, whereas high and extremely high nitrogen application (2.7 and 8.1 g/kg) significantly increased the population size of aphids. Both high and low nitrogen application benefited aphid growth in multiple aspects such as prolonging nymph period and adult lifespan, enhancing fecundity, and improving adult survival rate by elevating soluble sugar content in host Bt cotton plants. Cotton leaf Bt toxin content in medium nitrogen group (0.9 g/kg) was significantly higher than that in high (2.7 and 8.1 g/kg) and low (0.3 g/kg) nitrogen groups, but Bt toxin content in aphids was very low in all the nitrogen treatment groups, suggesting that medium level (0.9 g/kg) might be the optimal nitrogen fertilizer treatment level for promoting cotton seedling growth and inhibiting aphids.

**Discussion:**

Overall, this study provides insight into trophic interaction among nitrogen fertilizer levels, Bt cotton, and cotton aphid, and reveals the multiple effects of nitrogen fertilizer levels on growth and development of cotton and aphids. Our findings will contribute to the optimization of the integrated management of Bt cotton and cotton aphids under nitrogen fertilization.

## Graphical Abstract

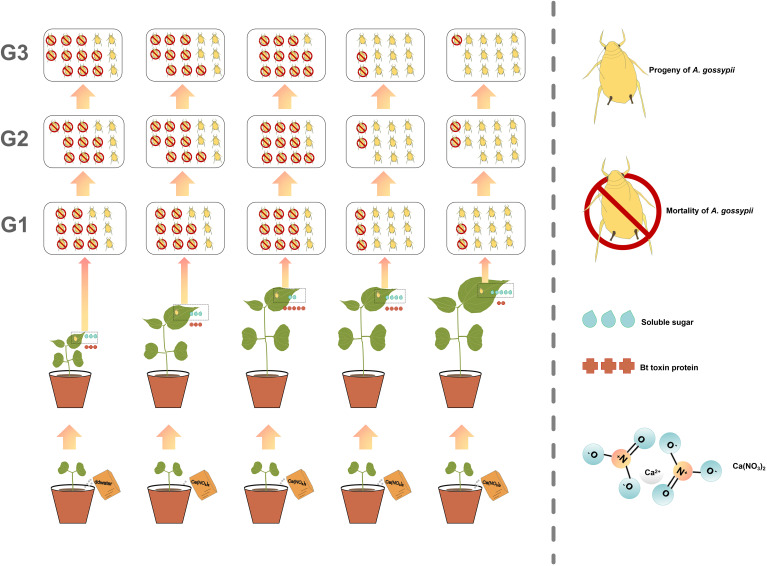

## 1 Introduction

Crops are vulnerable to various biotic and abiotic stresses at every stage of their growth (Jichao et al., 2021; Huangfu et al., 2022). Nitrogen (N) plays vital roles in plant growth and physiology (Seleiman et al., 2013; Eid et al., 2020). The total consumption of synthetic nitrogen fertilizer in China increased from 9.3 Mt in 1980 to 24 Mt in 2012 (Li et al., 2016). As a valuable commodity, cotton is widely cultivated around the world for the production of fiber, fuel, etc (Zafar et al., 2023). N lack or N excess will affect the growth and yield of cotton (Gerik et al., 1998). The nitrogen shortage in cotton plants will lead to the retarded growth of cotton and the decline in cotton fiber strength and quality, leaf area, stem length, biomass, photosynthetic efficiency (Zhao et al., 2003; Read et al., 2006; Zhang et al., 2008; Kakar et al., 2012). Nitrogen deficiency also brings about the decrease in cotton boll formation rate, thus reducing cotton yield (Lokhande and Reddy, 2015). In contrast, overdose of nitrogen fertilizer increases boll rot, decreases fiber length and strength, prolongs vegetative growth, delays cotton ripening, and potentially reduces lint yield (Zhang et al., 2008). The intensive use of nitrogen fertilizer in the agricultural fields directly increases the agricultural production cost. In addition, the application of a large level of nitrogen fertilizer causes a series of environmental problems such as groundwater pollution and soil acidification (Cameron et al., 2013; Iqbal et al., 2020; Seleiman et al., 2021; Al-Suhaibani et al., 2021; Elshayb et al., 2022; Shao et al., 2023). It has been reported that over-application of N fertilizers has caused significant soil acidification in major Chinese croplands and the soil pH decrease by 0.13 to 2.20 (Miao et al., 2011; Alkharabsheh et al., 2021), reasonable nitrogen application can make crop growth better (Gul Roz Khan et al., 2022).

Aphids are worldwide agricultural pests, which harm a variety of crops and cause huge economic losses (Quan et al., 2019). The cotton aphid, *Aphis gossypii* Glover (Hemiptera: Aphididae), an important pest on cotton, which can damage the seedling, buds, flowers, and bolls of cotton (Fernandes et al., 2012a). Cotton is widely used in the textiles and other industries in China (National Cotton Council 2006, http://www.cotton.org/) (Ruan, 2013; Jiang et al., 2019). Presently, cotton aphid is the main pest in China’s cotton fields, which has caused great harm to China’s cotton cultivation and reduced cotton yield (Wu and Guo, 2003; Wang et al., 2016; Jiang et al., 2019). However, there are few effective insecticides controlling the outbreak of cotton aphid. Recently, the combination of thiamethoxam and deltamethrin exhibits inhibitory effect on the life expectancy and reproductive rate of aphids, and plays a certain role in the prevention and control of cotton aphid (Majidpour et al., 2020). The application of sulfoxaflor through drip irrigation in Xinjiang province has been found to prolong the control period of cotton aphid, and it is relatively safety to natural enemies such as ladybird beetle and lacewing (Jiang et al., 2019). However, over-reliance on pesticides results in the resistance of cotton aphid to a variety of chemical pesticides (Morando et al., 2021). For example, a field *A. gossypii* Kushima withβ1 subunit R81T mutation in the nicotinic acetylcholine receptor (nAChR) exhibits high resistance to imidacloprid (Hirata et al., 2017). Cotton aphid has been found to have a strong resistance to fenvalerate, imidacloprid, and acetamiprid (Wang et al., 2007). The resistance of cotton aphid to various pesticides increases the difficulty and costs of controlling it.

Notably, the nitrogen fertilizer, as a nutrient of plants, can also trigger bottom-up plant-insect interaction effects (Mattson, 1980; Blazhevski et al., 2018; Ye et al., 2018). Nitrogen fertilizer can change the quality and quantity volatile chemicals in tomato plants, and high-level nitrogen fertilizer makes tomato plants release less volatiles and attract more *Bemisia tabaci* than normal level (10mM) or below normal level (5mM) of nitrogen fertilizer (Islam et al., 2017). High-level nitrogen fertilizer input in rice has unforeseen effects on crop susceptibility to pests, and increases adaptability of the brown planthopper *Nilaparvata lugens* (Rashid et al., 2017), High-level nitrogen fertilizer also raises the physiological indexes of rice, thus increasing colonization, survival, and development of the white-black planthopper *Sogatella furcifera* fed on rice (Li et al., 2021). In addition, the application of high-level nitrogen fertilizer influences the tritrophic interactions (crop-pest-natural enemy), significantly enhancing the abundance of three cereal aphids (namely *Sitobion avenae*, *Schizaphis graminum*, and *Rhopalosiphum padi*). Therefore, optimization of nitrogen fertilizer application is essential for crop planting. However, the knowledge about the effects of nitrogen fertilization levels on physiological and biochemical indexes of cotton and adaptability of cotton aphid remains limited.

Since 1996, the number of genetically modified (GM) crops has increased rapidly, especially the insect-resistant transgenic crops that produce the toxin (a insecticidal protein originally synthesized by *Bacillus thuringiensis* (Bt) bacteria) (James et al., 2009). The planting of Bt cotton has greatly reduced the application of pesticides in the field and effectively controlled many important lepidoptera pests (Naranjo, 2011). Compared with non-Bt cotton, Bt cotton can better control various pests such as *Spodoptera exigua*, *Helicoverpa armigera*, and *S. litura*, thus indirectly stimulating their natural enemy insect *Chrysopa* spp. to prey on more prey (Guan et al., 2022) due to the reduction in insecticide application. Although Bt planting can effectively reduce the target pests, sap-sucking insects such as cotton aphid, as non-target pests, have become the main pests with a trend of aggravation (Lu et al., 2012). Bt cotton can significantly alter spatiotemporal distribution pattern of cotton aphid inside the plant, with the development of the cotton (Fernandes et al., 2012b). The nitrogen application can significantly affect aphid population number, thus influencing cotton yield (Men et al., 2004). However, the mechanism of the effect of nitrogen application level on Bt cotton and *Aphis gossypii* is still unclear.

The nitrogen fertilizers in farmland mainly include ammonia nitrogen, nitrate nitrogen, and amide nitrogen. Especially, calcium nitrate (Ca(NO_3_)_2_), as one of the most valuable fertilizers on the market, can quickly supplement calcium and nitrogen, meanwhile improving fertilizer comprehensive utilization efficiency, eventually raising quality of crops and fruits (Colpaert et al., 2021; Tan et al., 2021). In laboratory experiments, Ca(NO_3_)_2_ is often utilized as nitrate nitrogen fertilizer to test nitrogen utilization efficiency in plants (Iqbal et al., 2020; Ochieng’ et al., 2023). In this study, Ca(NO_3_)_2_ was used as the only nitrogen source for cotton planting experiments. We investigated the effects of different nitrogen fertilizer levels on physiological and biochemical indexes of Bt cotton and adaptability and population number of cotton aphid (*Aphis gossypii)* by determining physiological and biochemical indexes determination of Bt cotton and comparing biological parameters of cotton aphid population under the treatment with different levels of nitrogen fertilizer. Our findings provide an insight into the interaction among the nitrogen fertilizer levels, Bt cotton, and cotton aphids, which will contribute to the optimization of Bt cotton-cotton aphid integrated management under nitrogen fertilizer application.

## 2 Materials and methods

### 2.1 Insect rearing and cotton planting

Cotton aphids were collected from the experimental cotton field (34.8^°^ N, 113.5^°^ E) of Institute of Cotton Research (ICR), Chinese Academy of Agricultural Sciences (CAAS) in Zhengzhou, and they were cultured on J14 cotton seedlings at 26 ± 1°C and 60 ± 5% room humidity (Light: Dark = 16: 8) in the laboratory. The cotton used in this study was insect-resistant transgenic cotton N15-5 (containing Cry2Aa toxin protein).

### 2.2 Setting of nitrogen fertilizer levels

According to the soil available nitrogen content in Xinjiang, China (Yang et al., 2012), a “soil N-Bt cotton-cotton aphid” system was established with different nitrogen fertilizer levels: control (0.0 g/kg), low (0.3 g/kg), medium (0.9 g/kg), high (2.7 g/kg), and extremely high (8.1 g/kg). Cotton seeds were sown in pot containing vermiculite, based on the weight of the substrate (vermiculite) for planting cotton (Vermiculite: solution = 1.05 g: 1 mL), the nitrogen fertilizer gradient was added to the quantitative nitrogen-deficient Hoagland solution. When cotton cotyledons were fully unfolded, the experiments started, and one cotton seedling was planted in one pot. Ca(NO_3_)_2_ was fertilized at different application levels of 0.0 g/kg, 0.3 g/kg, 0.9 g/kg, 2.7 g/kg, and 8.1 g/kg, with 0.0g/kg used as control. The CaCl_2_ was added to equilibrate the concentration of Ca^2+^ among different treatments (Iqbal et al., 2020). On the 7^th^, 14^th^, and 30^th^ day post cotton seedling growth, 10 cotton seedling plants were randomly selected from each experimental group for further analysis.

### 2.3 Measurement of leaf area and plant height

The length and width of leaves were measured, based on which leaf area was calculated using the coefficient method (Iqbal et al., 2019). The leaf area of two cotton cotyledons was measured on day 7 post nitrogen fertilization, while the leaf area of the first leaf (along the cotton plant counted from the top down) was calculated on day 14 and 30 post nitrogen ferelization, respectively. The stem length is measured through a ruler, as previously described (Shao et al., 2016; Alhammad et al., 2023).

### 2.4 Determination of Cry2Aa toxin and soluble sugar contents in cotton

Bt toxin protein content and soluble sugar content in the first cotton leaves were detected using the QuantiPlate Kit for Cry2Aa (EnviroLogix, Portland, USA) and Plant Soluble Sugar Content Assay Kit (Solarbio, Beijing, China), respectively. Each treatment was performed with three replicates.

### 2.5 Determination of Cry2Aa toxin protein content in *A. gossypii*

On day 14 post nitrogen fertilizer treatment, the first leaf (counted from the top) was picked from the cotton plant and put into the Petri dish (9 cm diameter, 2 cm height) with petiole inserted into 1.8% agar medium (prepared with18g of agar added into 1L of water), and 10-20 adult aphids were placed on one leaf to produce nymphs. After 24 hours, the adult aphids were removed, leaving 10-15 nymphs on each leaf. When nymphs grew into adults, they were collected and the Bt toxin protein in their body were detected according to the instruction of QuantiPlate Kit for Cry2Aa (EnviroLogix, Portland, USA). The Cry2Aa toxin determination was repeated three times.

### 2.6 Effects of nitrogen fertilizer levels on *A. gossypii* development and fecundity

Cotton plants treated with different levels of Ca(NO_3_)_2_ for 14 days were randomly selected. The first leaf (counted from the top) of the selected cotton plants was picked and inserted into the 1.8% agar medium in Petri dishes. Four adult cotton aphids were put on each leaf, and 24 h later, the adults were removed, leaving only one nymph offspring on the leaf. Subsequently, these nymphs were transferred onto new cotton leaf every 3-5 days and monitored on daily basis. The development period of the 1^st^ generation nymphs (G1) and offspring number produced by G1 were recorded daily. The identical experiments were performed on the second and third generation of cotton aphids (G2 and G3) fed on cotton seedlings treated with Ca(NO_3_)_2_ at different levels. There were 30 replicates under each nitrogen fertilizer level treatment.

### 2.7 Effects of nitrogen fertilizer levels on *A. gossypii* survival

On day 14 post nitrogen fertilizer treatment, the first leaf was inserted into the 1.8% agar medium, and 10-20 adult aphids were placed on each leaf to produce nymphs. Twenty four hours later, the adults were removed, leaving 10 nymphs on each leaf. These nymphs were observed, and their survival rate were recorded every day. The cotton leaves were replaced every 3-5 days. The experiment lasted for 15 days. The influence of nitrogen fertilizer levels on G2 and G3 of *A. gossypii* was evaluated, as above mentioned. There were 6 replicates for each treatment.

### 2.8 Effect of nitrogen fertilizer levels on *A. gossypii* population

On day14 post nitrogen fertilizer treatment, the first leaf was picked and inserted into the 1.8% agarose medium. Afterwards, 10-20 adult aphids were placed onto each leaf to produce nymphs, and 24 hours later, the adults were removed, leaving 10 nymphs on each leaf. These nymphs were observed, and their numbers were recorded on daily basis for 30 days. There were 3 replicates for each nitrogen fertilizer treatment.

### 2.9 Statistical analysis

Statistical analysis was performed with Microsoft Excel and SPSS (IBM SPASS Statistics 24). GraphPad Prism 8 was used for plotting. The statistical significance of differences between samples was analyzed using One-way ANOVA. All the data were expressed as mean ± standard error (SEM) of at least three biological replicates. The Kaplan-Meier survival curve was plotted by SPASS (IBM SPASS Statistics 24).

## 3 Results

### 3.1 Effects of nitrogen fertilizer levels on cotton plant height and leaf areas

**Figure 1:**
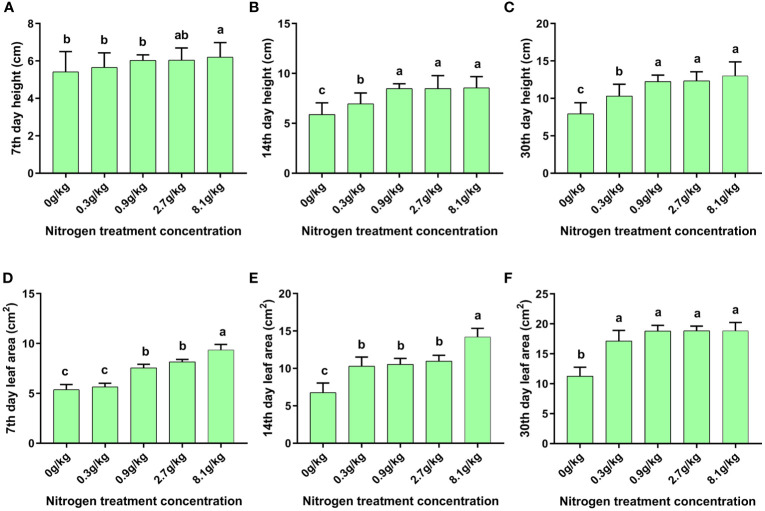
Morphological parameters of cotton seedlings. Agronomy parameters of N15-5 cotton seedlings in plant height and leaf area index at 7^th^ day **(A, D)**, 14^th^ day **(B, E)**, and 30^th^ day **(C, F)** post nitrogen fertilization under serial concentration gradients. The values are presented as mean ± SEM. Different lowercase letters (a, b, c, d) indicate that there are significant differences between the groups at the *p* < 0.05 level.

The cotton plant height and leaf area were measured at the 7^th^, 14^th^ and 30^th^ day after nitrogen treatments, respectively. Obviously, nitrogen fertilizer promoted the growth of cotton. At the 7^th^ (**Figure 1A**), 14^th^ (**Figure 1B**) and 30^th^ (**Figure 1C**) day post exposure to gradient nitrogen fertilizer, cotton height increased gradually with the increase of nitrogen fertilizer levels. Interestingly, there were no significant differences in plant height at the14^th^ and 30^th^ day among the cotton seedlings treated with relatively high fertilization levels of 0.9 g/kg, 2.7 g/kg, and 8.1 g/kg, but the plant height in these 3 relatively high level groups was all significantly higher than that in 0.3 g/kg and 0.0 g/kg nitrogen fertilizer groups (**Figures 1 B, C**). Similarly, the leaf area was also increased gradually with the rise of nitrogen fertilization levels (**Figures 1D–F**). Notably, in early stage of nitrogen fertilization (on day 7), significant differences in the leaf area were observed between treatment groups and control group, but no significant differences in plant height (**Figures 1A, D**), indicating that cotton leaf area responded to nitrogen fertilization more rapidly than plant height. However, on day 30 post nitrogen fertilization, no significant differences in leaf area were observed among the groups of 0.9 g/kg, 2.7 g/kg, and 8.1 g/kg (**Figure 1F**), and similar results were found in plant height (**Figure 1C**). Taken together, nitrogen fertilizer could promote the cotton growth, but the promotion effect was weakened under high nitrogen levels.

### 3.2 Effects of nitrogen fertilizer levels on Cry2Aa toxin protein content in cotton and *A. gossypii*

**Figure 2:**
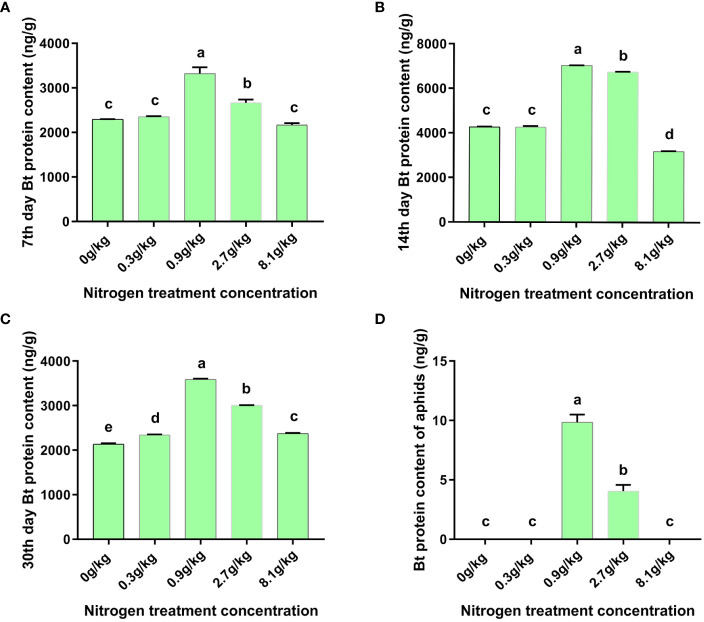
Content of Bt toxin protein in cotton leaves and *A gossypii*. Dynamics of Bt protein content in cotyledons of N15-5 seeding responding to nitrogen fertilization under serial concentration gradients at 7^th^ day **(A)**, 14^th^ day **(B)** and 30^th^ day **(C)** after nitrogen fertilization. Bt protein content **(D)** in cotton aphid feeding on N15-5 seeding leaves for 14 days post nitrogen fertilization under serial concentration gradients. The values are presented as mean ± SEM. Different lowercase letters (a, b, c, d, e) indicate that there are significant differences between the groups at the *p* < 0.05 level.

The influence of nitrogen fertilizer levels on Bt toxin content in cotton seedling was obvious. The Cry2Aa toxin content in the 0.0 g/kg, 0.3 g/kg, 8.1 g/kg groups was significantly lower that in 0.9 g/kg and 2.7 g/kg groups at all 3 time points (day 7, 14, and 30) after treatments (**Figures 2A–C**). Specifically, at day 7, 14, and 30, the Cry2Aa toxin content in cotton reached the peak in the medium level (0.9 g/kg) group, which was 3320.99 ng/g on day 7, 7045.51 ng/g on day14, and 3588.19 ng/g on day 30, followed by high level (2.7 g/kg) group, which was 2662.58 ng/g on day 7, 6752.95 ng/g on day 14, and 3008.71 ng/g on day 30. The extremely high nitrogen level (8.1 g/kg) inhibited the Cry2Aa content in cotton seedings at all test time points (2618.21 ng/g on day 7, 3182.67 ng/g on day 14, and 2376.19 ng/g on day 30). Overall, with the development of cotton seedlings, the Bt toxin content in all five groups exhibited the similar change pattern, namely, first elevation, and then reaching peak, followed by reduction, and Bt toxin content on day 30 was close to that on day 7.

The aphids were fed with the first leaves (counted from the plant top) after 14 days of nitrogen fertilizer treatment. The Cry2Aa toxin was detected in aphid individuals fed on cotton treated with 0.9 g/kg and 2.7 g/kg nitrogen fertilizer, which was 9.85 ng/g and 4.05 ng/g in these two groups (**Figure 2D**). Unexpectedly, Cry2Aa was not detected in the aphids fed on cotton seedlings exposed to control (0.0 g/kg), low (0.3 g/kg), extremely high (8.1 g/kg) nitrogen fertilizer, which might be due to relatively low Cry2Aa toxin levels in plants. In addition, the transmission efficiency of Cry2Aa between cotton and aphids was very low, with the transmission efficiency below 0.3% (10 ng/g) under all nitrogen fertilizer levels.

### 3.3 Effects of nitrogen fertilizer levels on cotton soluble sugar content and *A. gossypii* population

**Figure 3:**
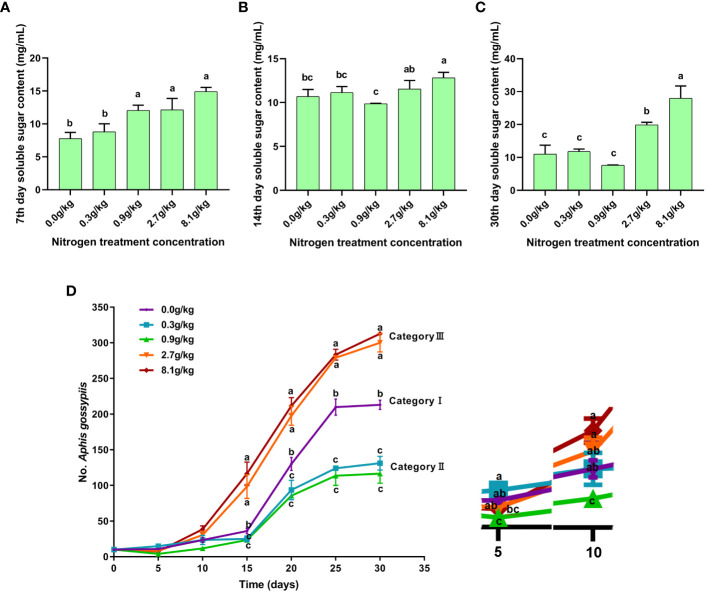
Soluble sugar content at 7^th^ day **(A)**, 14^th^ day **(B)** and 30^th^ day **(C)** post nitrogen fertilization under serial concentration gradients. Population dynamics of *A gossypii* feeding on cotton seedlings as description above for thirty days **(D)**. The values are presented as mean ± SEM. Different lowercase letters (a, b, c) indicate that there are significant differences between the groups at the *p* < 0.05 level.

The nitrogen fertilizer levels heavily influenced the soluble sugar content in Bt cotton plant. On the 7^th^ day after treatment, the soluble sugar content in cotton leaves in the control (0.0 g/kg) and low (0.3 g/kg) nitrogen fertilizer groups was significantly lower than that in the medium (0.9 g/kg), high (2.7 g/kg), and extremely high (8.1 g/kg) nitrogen fertilizer groups (**Figure 3A**). On the 14^th^ day after treatments, the cotton leaf soluble sugar content was significantly higher in control (0 g/kg), low (0.3 g/kg), high (2.7 g/kg), extremely high (8.1 g/kg) nitrogen groups than in medium nitrogen group (0.9 g/kg) (**Figure 3B**). More specifically, on the 14^th^ day after treatments, the leaf soluble sugar content in low nitrogen group (11.13 mg/mL) was slightly higher than that in control group (10.69 mg/mL), and medium nitrogen group exhibited the minimum leaf soluble sugar content (9.87 mg/mL). Leaf soluble sugar content in high and extremely high nitrogen groups was 17% and 30% higher than that in medium nitrogen group, respectively. On the 30^th^ day after treatments, the soluble sugar content in cotton leaves was significantly lower in the control, low, and medium nitrogen groups than in the high and extremely high nitrogen groups (**Figure 3C**). It was worth noting that the soluble sugar in the medium nitrogen group was the minimum (7.63 mg/mL), it was the maximum in the extremely high nitrogen group (27.96 mg/mL).

The aphids were fed with the first leaves of cotton plants treated with nitrogen fertilizer after 14 days, and their growth status was observed. The results showed that nitrogen fertilizer levels had obvious effect on aphid growth. The cotton aphid growth curves showed that cotton aphids clustered into three categories, namely, category I (0 g/kg nitrogen fertilizer group), category II (0.3 g/kg and 0.9 g/kg nitrogen fertilizer group), and category III (2.7g/kg and 8.1g/kg nitrogen fertilizer groups). Category III exhibited the highest growth rate, especially on day 15, 20, 25, and 30 after aphid feeding (**Figure 3D**). The differences in growth rate were significant among these three categories. In the early stage (5^th^ and 10^th^ day after aphid feeding), the cotton aphid populations in all three categories displayed a slow growth rate. Ultimately, on 30^th^ day, the cotton aphid population number in 8.1 g/kg, 2.7 g/kg, 0.0 g/kg nitrogen fertilizer groups reached 313, 300, and 213, respectively, which was significantly higher than that in 0.9 g/kg and 0.3 g/kg groups. Compared with the other four groups, the cotton aphids in 0.9 g/kg nitrogen fertilizer group displayed the lowest population growth rate in the six detection time points (0^th^, 5^th^,15^th^, 20^th^, 25^th^, and 30^th^).

### 3.4 Multi-generational effects of nitrogen fertilizer levels on the development and survival of *A. gossypii*

Nitrogen fertilization levels significantly influenced the duration time of nymph stage and fecundity of cotton aphid across three generations, but had no significant effect on the lifespan of adults (**Table 1**). Compared to control (0.0 g/kg), low (0.3 g/kg), high (2.7 g/kg), extremely high (8.1 g/kg) nitrogen fertilizer groups, cotton aphids fed on cotton leaves treated with medium nitrogen (0.9 g/kg) had the shortest duration time of nymph stage across three generations with a continuously shortening trend. In the five nitrogen treatment groups, the extremely high nitrogen group had the longest nymphal development period in all three generations. High (2.7 g/kg) and extremely high (8.1 g/kg) nitrogen fertilization promoted the fecundity of cotton aphids in all three generations. Especially in the extremely high nitrogen (8.1 g/kg) treatment group, the number of cotton aphids increased from 42 in the first generation to 47 in the second generation, and reached 58 in the third generation. Although low (0.3 g/kg) nitrogen fertilization had no significant influence on cotton aphid fecundity at the 1st generation, the offspring number of 2^nd^ and 3^rd^ generations was obviously larger than that in control group.

**Table 1:** Life cycle of *A. gossypii*.

		Nitrogen treatment concentration				
Generation		0 g/kg	0.3 g/kg	0.9 k/kg	2.7 g/kg	8.1 g/kg
**G1**	Days of nymphs	6.20 ± 2.56ab	5.80 ± 3.12ab	4.10 ± 2.55b	5.40 ± 2.42ab	7.10 ± 1.92a
	Days of lifespan	35.00 ± 1.00ns	30.50 ± 4.61ns	25.50 ± 7.43ns	28.50 ± 0.87ns	30.33 ± 4.89ns
	No. of offspring	30.00 ± 3.56bc	28.25 ± 4.49c	17 ± 0.71d	36.50 ± 4.50ab	42.00 ± 5.40a
**G2**	Days of nymphs	4.10 ± 1.87bc	5.50 ± 1.43ab	3.30 ± 1.10c	5.10 ± 1.45ab	5.70 ± 1.19a
	Days of lifespan	18.00 ± 0.82ns	20.67 ± 3.30ns	19.00 ± 0.82ns	22.33 ± 1.70ns	22.00 ± 2.94ns
	No. of offspring	34.00 ± 1.00c	42.50 ± 4.50ab	18.33 ± 0.47d	40.33 ± 2.05ab	47.33 ± 4.92a
**G3**	Days of nymphs	5.52 ± 1.03b	6.11 ± 0.74b	2.89 ± 1.59c	6.44 ± 1.17ab	7.44 ± 1.07a
	Days of lifespan	18.83 ± 5.73ns	21.71 ± 5.17ns	21.71 ± 4.65ns	22.00 ± 3.61ns	21.00 ± 2.61ns
	No. of offspring	34.60 ± 11.25c	41.33 ± 13.17ab	26.00 ± 5.20c	54.38 ± 7.09a	58.00 ± 15.99a

The cotton aphid nymphic duration times, adult lifespan and fecundity across successively three generations when fed on N15-5 cotton under nitrogen fertilizer of serial concentration gradients. The values are presented as mean ± SEM (the first generation G1; the second generation G2; the third generation G3). Different lowercase letters (a, b, c, d) indicate that there are significant differences between the groups at the *p* < 0.05 level, and ns indicates that there is no significant difference between the groups.

Interestingly, the fecundity of cotton aphids across successive three generations (G1, G2, and G3) in medium nitrogen group (0.9 g/kg) was all significantly inhibited fertilization), in comparison to control, low, high, and extremely high nitrogen groups. The fecundity of cotton aphids in all five nitrogen treatment groups was all gradually increased across the three generations. The medium nitrogen fertilization obviously suppressed the reproduction of cotton aphids, whereas the extremely high nitrogen fertilization significantly promoted the fecundity of cotton aphids (**Table 1**).

**Figure 4:**
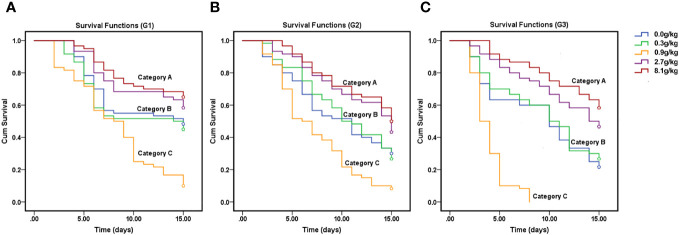
The survival rate of *A. gossypii.* Survival rate of cotton aphid population across successively three generations within 15 days feeding on N15-5 cotton seedling (the first generation G1, **(A)** the second generation G2, **(B)** the third generation G3, **(C)**).

Our data also showed that nitrogen fertilizer levels had significant effects on the survival rate of aphids (**Figures 4A–C**). The cotton aphid survival rate curves across three generations showed that cotton aphids clustered into three categories, namely, Category A (8.1 g/kg and 2.7 g/kg nitrogen fertilization groups), Category B (0 g/kg and 0.3 g/kg nitrogen fertilization group), Category C (0.9 g/kg nitrogen fertilization group). Category A exhibited the highest survival rate of cotton aphids across successive three generations, followed by Category B, and Category C had the lowest survival rate of cotton aphids. Cotton aphid survival rate curves showed that in G1, the three categories obviously differentiated from 7^th^ day, whereas in G2 and G3, they obviously differentiated from 4^th^ day. In G3, the aphid survival rate in medium nitrogen fertilization group reduced sharply to 10% within five days after aphid feeding, suggesting an obvious aphid controlling effect.

## 4 Discussion

**Figure 5:**
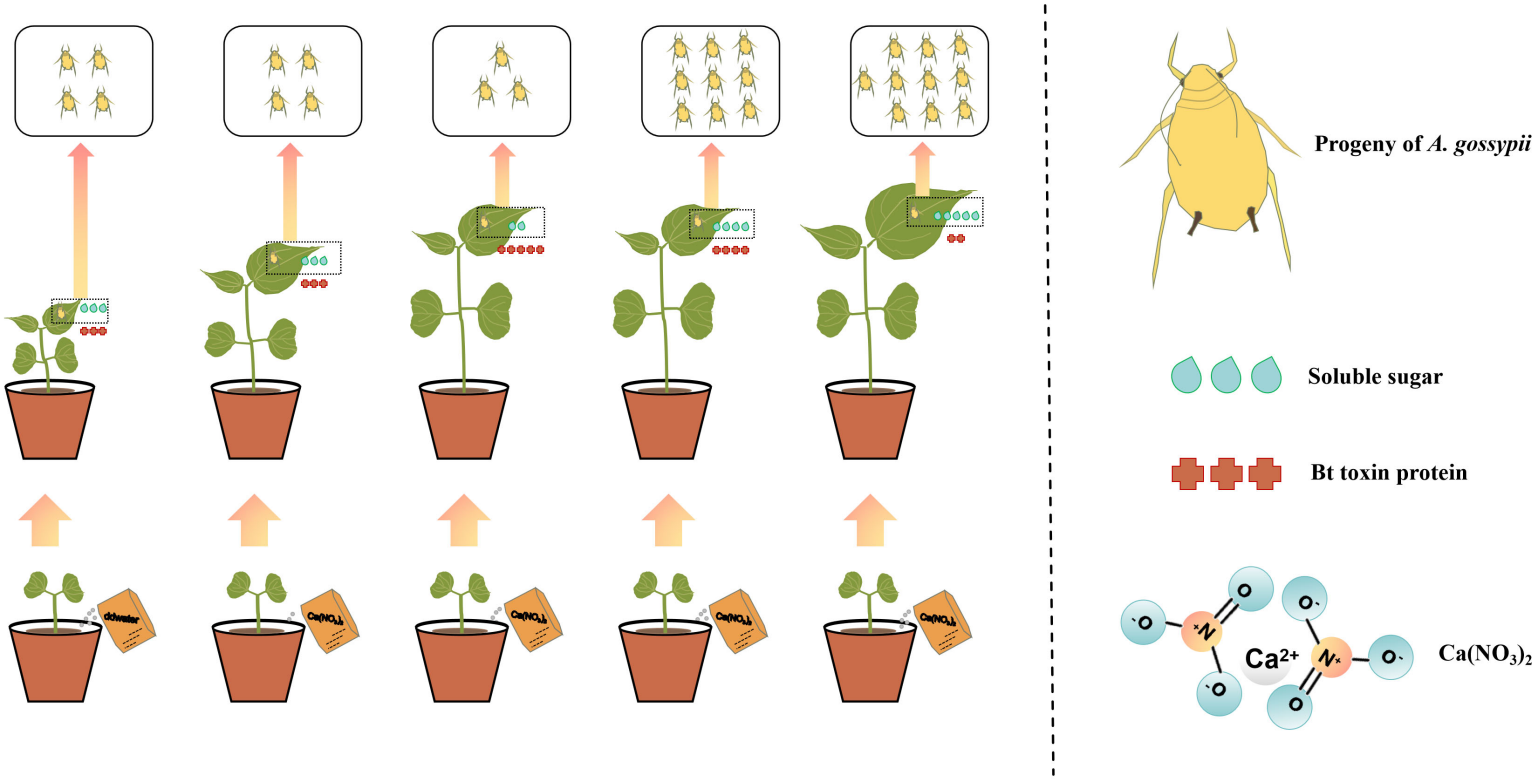
Interaction patterns of Bt cotton and *A. gossypii* under different nitrogen fertilizer levels.

Evidence is mounting that nitrogen fertilizer is an important factor affecting plant growth and development in agricultural planting system. This study revealed the interactions among different nitrogen fertilizer levels (0.0 g/kg, 0.3 g/kg, 0.9 g/kg, 2.7g/kg, 8.1g/kg), Bt cotton, and cotton aphid (**Figure 5**). All the plants exhibit some morphological changes under different levels of nitrogen fertilization (Sakakibara et al., 2006). Our one-way ANOVA analysis showed that there were significant differences in cotton plant height and leaf area under different nitrogen fertilizer levels at the seedling stage, which was consistent with previous reports (Iqbal et al., 2020; Wang et al., 2020b). Plant height and leaf area will affect photosynthesis of crops, thus affecting the growth and development of crops (Hutchison et al., 1990; Chen et al., 2023). In our experiment, the plant height and leaf area of cotton plants after nitrogen application were higher than those without nitrogen application. With the extension of nitrogen application time, the difference of plant height between different treatment groups became more and more obvious (**Figure 1**), indicating that nitrogen fertilizer can indeed affect the growth of cotton plants at seedling stage, and this change will be more obvious with time. And with the change of nitrogen application level, the plant height and leaf area of cotton plants also changed. As for how nitrogen fertilizer affects plant growth and development through molecular level, we need to further study.

Nitrogen fertilizer levels influence herbivorous insects. The application of high nitrogen fertilizer increased the feeding ability and fecundity of crop pests (Minkenberg and Ottenheim, 1990), Besides, the application of high nitrogen fertilizer also can increase the feeding ability and fecundity of crop pests (Hu et al., 2022), which caused serious harm to the growth and yield of crops. In our study, the growth and development of aphids were significantly altered by the application of different levels of nitrogen fertilizer (Carreras Navarro et al., 2020; Wang et al., 2020a). Interestingly, the aphid population number in medium nitrogen treatment group was the smallest, even smaller than that in control group and low nitrogen group. However, when the nitrogen fertilizer level was higher than 0.9 g/kg, the aphid population number was sharply increased (**Figure 3D**). In the three generations, cotton aphids in medium nitrogen group exhibited the shortest duration of nymph stage and the shortest adult lifespan, and the offspring number was also the smallest in this medium nitrogen group (**Table 1**). However, high nitrogen and extremely high nitrogen groups exhibited a had relative longer nymph period and a larger offspring number. In addition, aphids fed on cotton leaves treated with medium nitrogen level had the lowest adult survival rate, while those treated with high nitrogen and extremely high nitrogen exhibited the highest adult survival rate. Considering this, excessive application of nitrogen fertilizer not only prolongs the damage time of cotton aphids, but also increases the population of cotton aphids, which will aggravate the damage of cotton aphids to the growth and development of cotton plants at the seedling stage. The medium level of nitrogen fertilizer is the best nitrogen application level to inhibit the damage of cotton aphids, which is extremely beneficial to the development of cotton plants at the seedling stage.

Nitrogen fertilization can bring about changes in the content of Bt toxin protein. It has been reported that the application concentrations of nitrogen and phosphate fertilizers are positively correlated with the expression of Cry1Ac protein in Bt cotton (Khan et al., 2021), which was different from our results. In this study, the Cry2Aa protein content in N15-5 cotton did not always rapidly rise with the increase in nitrogen fertilizer level. In contrast, the Bt protein content of cotton peaked at 0.9g/kg nitrogen fertilizer level, but subsequently dropped obviously with the elevation of nitrogen fertilizer levels (**Figures 2A-C**). It is well known that transgenic Bt cotton can effectively inhibit various target pests (Kranthi and Stone, 2020; Quan and Wu, 2023). Therefore, for the target pests of N15-5 transgenic insect-resistant cotton, we believe that the control effect of medium nitrogen fertilizer level (0.9 g/kg) will be better and with the increase of nitrogen fertilizer dosage, the decrease of Bt protein may inevitably lead to the rampant of Bt cotton target pests. In addition, the study found that the content of Bt protein in cotton had no significant effect on aphids (Zhao et al., 2016). In this study, the efficiency of Cry2Aa toxin protein transfer from cotton leaves to aphids was extremely low at all nitrogen levels (**Figure 2D**), based on this result, we speculate that the change of Bt protein content affected by nitrogen fertilizer may have no effect on the natural enemies of cotton aphids such as ladybugs. Besides, our data also showed that there was no close correlation between the changes in aphid biological parameters and the dynamic trend of Bt protein content. The mechanism of nitrogen fertilizer affecting the growth and development of aphids needs to be further studied, so we detected metabolites such as soluble sugar in cotton leaves for further exploration.

The changes of metabolites in crops affect the growth and development of crops. Soluble sugar is an important indicator of physiological changes in cotton. The change of fertilizer dosage can change the soluble sugar content in cotton plants (Hu et al., 2017) (Pan et al., 2011; Li et al., 2017, 2019). Metabolites such as soluble sugars have an impact on crop pests (Singh et al., 2022).In our experiment, soluble sugar contents varied with the nitrogen fertilizer levels, which is consistent with previous research. Notably, the soluble sugar content in cotton leaves reached its lowest level under medium nitrogen level, and then gradually increased under high and extremely high nitrogen levels (**Figures 3B, C**). Our data showed that the dynamics of soluble sugar content in cotton (**Figure 3B**) are consistent with the changes of cotton aphid growth and development (**Table 1**), and the soluble sugar content was highly expressed at high and extremely high nitrogen levels (**Figure 3B**), and the population of *A.gossypii* also expanded rapidly (**Figure 3D**). It is reasonable to think that nitrogen fertilizer can change the growth and development of cotton aphid by changing the content of soluble sugar in cotton plant.

## 5 Conclusion

This study revealed that nitrogen fertilizer levels significantly affected the cotton morphology, and medium level of nitrogen fertilizer could make the cotton seedling grow more uniformly and promote the Bt toxin protein (Cry2Aa) content in Bt cotton. In contrast, high nitrogen fertilization reduced the content of Bt toxin protein. Different nitrogen fertilizer levels led to variations in the growth and development of cotton aphids by altering soluble sugar content in cotton plants. The content of soluble sugar, a metabolite of cotton plants, was increased in leaves at high and extremely high nitrogen levels, which led to the obvious increase in survival rate, reproductive capacity, and population number of *A. gossypii*. Under medium nitrogen level treatment, the leaf sugar content was relatively low, and the survival rate, reproductive capacity, and population number of aphids were reduced. Therefore, medium-level nitrogen fertilizer exhibited the optimal inhibitory effect on aphid growth and development. Overall, this study provides insight into trophic interaction among nitrogen fertilizer levels, Bt cotton, and cotton aphid, and demonstrates the a series of effects of nitrogen fertilizer levels on growth and development of cotton and aphids. Our findings will contribute to the optimization of the integrated management of Bt cotton and cotton aphid under nitrogen fertilization. Our findings will contribute to the sustainable development of cotton production and environmental management.

## Data availability statement

The original contributions presented in the study are included in the article/supplementary files. Further inquiries can be directed to the corresponding authors.

## Author contributions

LG: Writing – original draft, Writing – review & editing, Data curation, Formal analysis, Investigation, Methodology, Validation, Visualization. LN: Writing – review & editing, Formal analysis, Investigation, Methodology. XZ: Writing – review & editing, Investigation, Methodology. LW: Writing – review & editing, Investigation, Methodology. KZ: Writing – review & editing, Investigation, Methodology. DL: Writing – review & editing, Investigation, Methodology. PE: Writing – review & editing, Visualization. XG: Writing – review & editing, Data curation. JJ: Writing – original draft, Formal analysis, Project administration, Supervision, Visualization. JC: Writing – review & editing, Formal analysis, Project administration, Funding acquisition, Resources, Supervision. JL: Writing – review & editing, Formal analysis, Project administration, Funding acquisition, Resources, Supervision.
